# Supplementary material for: Real-World Safety and Effectiveness of Voretigene Neparvovec: Results up to 2 Years from the Prospective, Registry-Based PERCEIVE Study
Source: Biomolecules. 2024 Jan 17;14(1):122. doi: 10.3390/biom14010122 (PMC10813228; doi:10.3390/biom14010122)

Supplementary materials:

Supplementary Figure S1: Patient disposition flow chart

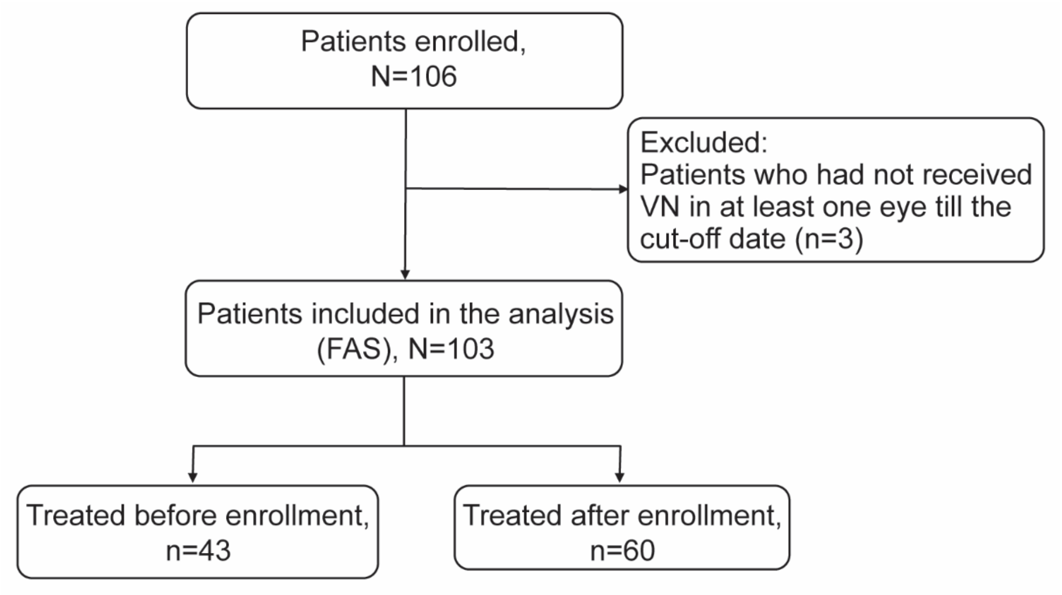

Supplementary Figure S2: Change in visual field (Octopus kinetic perimetry [V4e]) from baseline

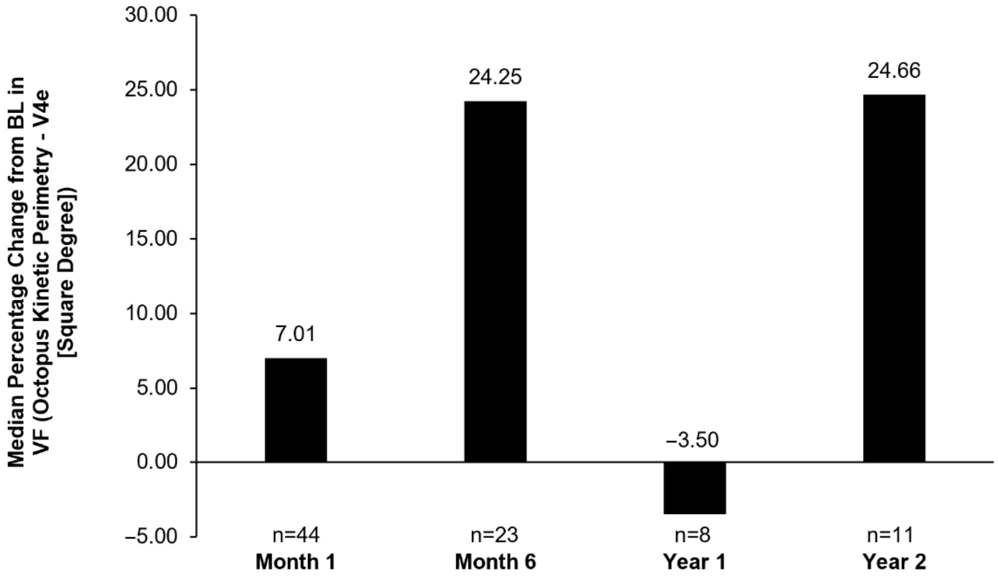

Supplement: Supplementary file 1 [file biomolecules-14-00122-s001.zip › biomolecules-2686746-supplementary.pdf]
